# OTULIN: a master regulator of linear ubiquitin homeostasis in immune signaling, inflammation, and disease

**DOI:** 10.3389/fimmu.2026.1906115

**Published:** 2026-08-05

**Authors:** Ziyu Zhang, Jiahang Lyu, Yujie Bai, Zhen Guo, Qifu Bo, Kai Liu, Xiangpeng Dai

**Affiliations:** 1 First Hospital of Jilin University, Changchun, Jilin, China; 2 Department of Comprehensive Oncology, Affiliated Hospital of Shandong Second Medical University, Weifang, China; 3 Key Laboratory of Organ Regeneration and Transplantation of Ministry of Education, First Hospital of Jilin University, Changchun, China; 4 National-Local Joint Engineering Laboratory of Animal Models for Human Disease, First Hospital of Jilin University, Changchun, China

**Keywords:** immune homeostasis, linear ubiquitination, LUBAC, NF-κB, OTULIN, targeted therapy

## Abstract

OTU deubiquitinase with linear linkage specificity (OTULIN) is the only deubiquitinase known to exhibit strict specificity for Met1-linked linear ubiquitin chains, serving as a critical regulator that restrains linear ubiquitin chain assembly complex and maintains inflammatory homeostasis and tissue integrity. Advances over recent years have reshaped our understanding of OTULIN from a negative regulator confined to the canonical nuclear factor kappa-light-chain-enhancer of activated B cells pathway to a central editor governing linear ubiquitin network homeostasis. Its functional repertoire spans receptor-proximal inflammatory signaling, cell death fate determination, inflammasome activation, autophagy, and cellular stress adaptation. Emerging evidence from human genetics and animal models has further broadened the OTULIN-associated disease spectrum, revealing that pathogenesis arises from imbalanced linear ubiquitin networks coupled with cell type and tissue context dependent inflammatory dysregulation. Here, we provide a comprehensive overview of OTULIN’s structural features, regulatory mechanisms, biological functions, roles in pathogenesis, and potential therapeutic intervention strategies, aiming to inform future mechanistic studies and facilitate translational advances in targeted therapies.

## 1 Introduction

The ubiquitin system orchestrates protein fate, signal transduction, and cellular adaptive responses through the assembly, recognition, and disassembly of topologically distinct ubiquitin chain types (1–3). Among these, Met1-linked linear ubiquitin chains, generated exclusively by the linear ubiquitin chain assembly complex (LUBAC), represent a critical non-degradative ubiquitin signal within innate immune and inflammatory signaling platforms, functioning primarily as molecular scaffolds that amplify downstream signaling (4–6). Unlike K48-linked chains, which predominantly target substrates for proteasomal degradation, or K63-linked chains, which serve as signaling scaffolds, Met1-linked chains act as highly organized molecular scaffolds that stabilize receptor-proximal signaling platforms at TNFR1, IL-1R/TLR, and NOD receptors, recruiting the NEMO/IKK complex, and establishing cell fate decision thresholds (5, 7–9). OTU deubiquitinase with linear linkage specificity (OTULIN) is the only deubiquitinase to exhibit strict specificity for Met1-linked chains, and its biological significance therefore extends well beyond the simple removal of excess ubiquitin. Rather than serving merely as a passive “housekeeper” that removes excess Met1-linked ubiquitin chains after signaling has subsided, OTULIN constitutively associates with HOIP through its PUB-interacting motif, thereby positioning it in close proximity to sites of linear ubiquitin chain assembly (10, 11). Coupled with its substrate-assisted catalytic activation upon recognition of Met1-linked ubiquitin chains, this spatial arrangement suggests that OTULIN may function as a dynamic editor of linear ubiquitin homeostasis (12, 13). By continuously constraining the abundance, persistence, and spatial distribution of linear ubiquitin signals, OTULIN helps preserve appropriate signaling thresholds, thereby restricting spurious inflammation and maintaining immune tolerance (14, 15). However, direct live-cell or chain-resolved evidence that OTULIN trims a defined substrate-bound Met1-linked chain in real time remains lacking.

Early studies defined OTULIN as a negative regulator of LUBAC, overexpression of OTULIN suppressed LUBAC driven Nuclear factor kappa-light-chain-enhancer of activated B cells (NF-κB) activation, whereas OTULIN deficiency resulted in aberrant linear ubiquitination of LUBAC components, prolonged survival of receptor signaling complexes, and amplified inflammatory output (12, 13). Accumulating evidence, however, has revealed that this framework is overly narrow. First, OTULIN participates not only in canonical inflammatory signaling but also in the regulation of cell death, proteostasis, autophagy, DNA damage responses, embryonic and vascular development (16–19). Second, OTULIN’s function is not equivalent across cell types such as myeloid cells, keratinocytes, hepatocytes, intestinal epithelial cells, and stromal cells which differ markedly in their sensitivity to linear ubiquitin imbalance, giving rise to tissue specific patterns of inflammation and injury (16, 20–23). Third, the OTULIN-associated disease spectrum constitutes a continuum shaped by the interplay of gene dosage, structural perturbation, protein–protein interactions, and environmental context, rather than conforming to a simple Mendelian paradigm (23, 24).

Accordingly, a conceptual framework centered solely on OTULIN as a negative regulator of NF-κB no longer captures the breadth of current knowledge. In this review, we adopt an enzymological and mechanistic biology perspective to systematically summarize and discuss the molecular basis of OTULIN as a linear ubiquitin-editing enzyme in regulating multiple signaling pathways and human diseases, and emerging therapeutic prospects to provide an integrated framework for future mechanistic investigation and clinical translation of OTULIN.

## 2 Structure of OTULIN: domains and related functions

**Figure 1:**
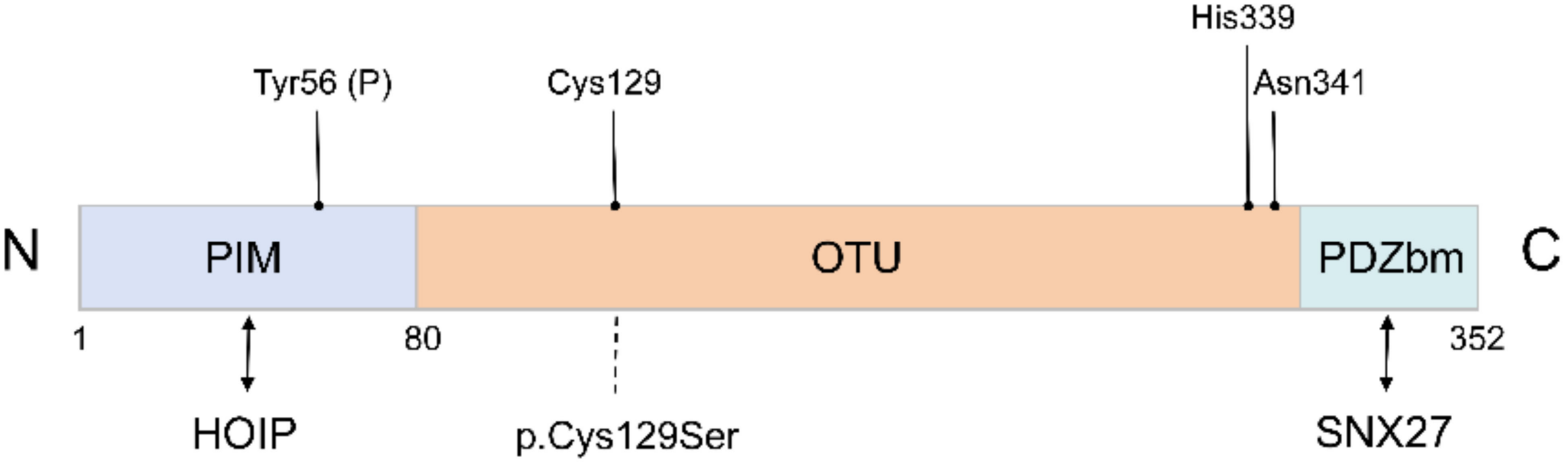
The basic structure of OTULIN. Human OTULIN comprises an N-terminal PUB-interacting motif, a central ovarian tumor catalytic domain, and a C-terminal PDZ-binding motif. The PUB-interacting motif mediates OTULIN binding to HOIP, whereas the PDZ-binding motif contributes to its interaction with SNX27. The Tyr56 phosphorylation site, catalytic residues Cys129, His339, and Asn341, and the disease-associated p.Cys129Ser mutation are indicated.

OTULIN (**Figure 1**) is a 352-amino-acid deubiquitinase belonging to the ovarian tumor (OTU) domain family. Unlike most OTU family members, which recognize multiple isopeptide bond linkages, OTULIN displays remarkably strict specificity for Met1-linked linear ubiquitin chains (12, 25). Structurally, OTULIN comprises three functionally distinct modules, including an N-terminal PUB-interacting motif (PIM), a central catalytic OTU domain, and a C-terminal PDZ-binding motif (PDZbm) (10, 26).

### 2.1 PUB-interacting motif

The PIM is a short linear motif located at the N-terminus of OTULIN. LUBAC, composed of HOIP, HOIL-1L, and SHARPIN, is the only E3 ligase complex definitively shown to catalyze Met1-linked chain assembly, with HOIP serving as the principal catalytic subunit (6, 27).

Rather than contributing to catalysis directly, the PIM mediates the interaction between OTULIN and the PUB domain of HOIP, forming a constitutive complex with LUBAC even under resting conditions and thereby recruiting OTULIN to the linear ubiquitin signaling axis (10, 11). This writer eraser coupling architecture positions OTULIN in immediate proximity to the chain generating machinery, enabling real-time trimming of nascent linear chains and preventing aberrant self-amplification of either LUBAC itself or receptor signaling complexes. It is this structural arrangement that establishes OTULIN as an integral regulatory component of the LUBAC complex.

### 2.2 OTU domain

The OTU domain, which residues 80–352, constitutes the core catalytic module of OTULIN and provides the structural basis for its exquisite Met1-linkage specificity (12).

OTU domain simultaneously engages both the proximal and distal ubiquitin moieties of a linear diubiquitin substrate, forming a highly selective substrate-binding interface that ensures precise cleavage of the Met1 linkage (28). The catalytic center is composed of a classical catalytic triad, including Cys129, His339, and Asn341, in which Cys129 functions as the nucleophilic attack site, His339 modulates the reactivity of Cys129 during catalysis, and Asn341 stabilizes the spatial organization of the active site to maintain the conformation required for chain cleavage (29). Notably, the catalytic triad does not adopt a fully competent conformation in the absence of substrate. Instead, binding of the cognate linear ubiquitin substrate triggers active-site rearrangement through the participation of Glu16 on the proximal ubiquitin, which promotes assembly of the catalytic triad into its activated state (12). This substrate-assisted catalytic mechanism not only accounts for the high catalytic efficiency of OTULIN toward Met1-linked chains but also provides a structural explanation for its absolute linkage specificity.

### 2.3 PDZ-binding motif

The C-terminal PDZbm mediates the interaction of OTULIN with sorting nexin 27 (SNX27). This interaction does not appreciably alter OTULIN’s catalytic activity, its association with LUBAC, or global Met1-linked ubiquitin levels in the cytoplasm. Instead, PDZbm binding to SNX27 primarily influences the assembly of the SNX27–retromer complex, cargo recognition, and receptor recycling (26). The PDZbm is therefore regarded as a structural determinant through which OTULIN participates in protein localization, endosomal sorting, and membrane protein trafficking, rather than a region essential for its basal deubiquitinase activity. The coexistence of catalysis-dependent and catalysis-independent functions exemplifies the emerging recognition of OTULIN as a multifunctional regulatory protein, one of the more representative examples of functional versatility within the deubiquitinase field in recent years (30).

## 3 Regulation of OTULIN: transcription, post-translational modifications

### 3.1 Transcriptional regulation

Available evidence indicates that OTULIN expression is neither uniform nor constitutive but rather exhibits pronounced tissue specificity, cell type selectivity, and context dependence.

Within the immune system, OTULIN is broadly expressed across T cells, B cells, natural killer (NK) cells, and myeloid lineages. However, its expression levels vary considerably among these populations, suggesting that basal transcriptional output is tightly coupled to lineage specific programs and homeostatic demands (14). Conditional knockout models in distinct organ systems reinforce this view, keratinocytes, endothelial cells, intestinal epithelial cells, and hepatocytes each displaying different degrees of dependence on OTULIN, indicating that its expression is not solely dedicated to inflammatory restraint but is also embedded within broader developmental and tissue homeostasis networks (20, 22, 31). Notably, under inflammatory and stress conditions, OTULIN does not follow a simple pattern of uniform upregulation or downregulation. Instead, its expression undergoes dynamic, stimulus dependent and cell type dependent fluctuations. In certain inflammatory diseases, diminished OTULIN expression has been linked to excessive inflammasome activation (32), whereas in selected malignancies and under therapeutic stress such as chemotherapy, OTULIN can be aberrantly upregulated and associated with adaptive tumor cell survival, drug resistance, or invasive phenotypes (33, 34). OTULIN expression is therefore shaped by the convergence of inflammatory milieu, tissue stress, and cellular differentiation state, rendering its regulation highly context specific. Although the context-dependent expression of OTULIN is increasingly appreciated, the molecular mechanisms governing its transcription remain largely elusive. In contrast to the deubiquitinases CYLD and A20, whose expression is transcriptionally induced through NF-κB-dependent negative-feedback circuits, no evidence has yet demonstrated that OTULIN is a direct transcriptional target of NF-κB, nor has a bona fide master transcription factor regulating OTULIN expression been identified (30). Consequently, the promoter architecture, cis-regulatory elements, transcription factor occupancy, and epigenetic mechanisms controlling OTULIN transcription remain largely unexplored, representing a major knowledge gap in the field and an important direction for future investigation.

### 3.2 Cellular location dependent regulation

OTULIN has traditionally been characterized as a cytosol resident deubiquitinase with strict specificity for Met1-linked ubiquitin chains. Emerging evidence, however, reveals that its function is not confined to the diffuse cytoplasmic environment but instead depends on conditional recruitment to specific signaling platforms and subcellular compartments (12, 30). In canonical inflammatory signaling, OTULIN is recruited to LUBAC associated complexes through the interaction of its N-terminal PIM with the PUB domain of HOIP. Upon formation of receptor signaling platforms such as the TNFR1 signaling complex (complex I), OTULIN gains access to the receptor-proximal vicinity, where it locally trims Met1-linked ubiquitin chains to restrain excessive NF-κB activation (11, 35). Beyond receptor signaling complexes, OTULIN participates in spatially distinct regulatory circuits. Its C-terminal PDZbm interacts with SNX27, modulating SNX27-retromer complex-mediated endosomal recycling and membrane protein trafficking, a finding that implicates OTULIN in non-canonical functions related to membrane protein sorting and intracellular transport (26). Furthermore, OTULIN deficiency has been associated with aberrant accumulation of linear ubiquitin chains on proteasome subunits, proteasomal dysfunction, and enhanced type I interferon responses, suggesting that its functional reach extends to the proteostasis machinery (19). Under genotoxic stress, OTULIN also modulates DNA damage-associated NF-κB responses and influences cell survival decisions (18).

Collectively, these observations establish that OTULIN does not operate exclusively within a single cytoplasmic milieu but rather distributes dynamically among receptor-proximal inflammatory platforms, the endosomal sorting system, and proteostasis-associated structures. A key unresolved question, however, is what governs OTULIN’s redistribution among these compartments, whether it is primarily dictated by LUBAC localization, *de novo* substrate chain formation, or the cooperative action of additional bridging proteins.

### 3.3 Post-translational modifications

The best characterized post-translational modification (PTM) of OTULIN is phosphorylation at Tyr56, a residue located in close proximity to the PIM (**Figure 1**). Tyr56 constitutes a critical contact point for the OTULIN–HOIP PUB domain interaction, and its phosphorylation weakens this association, thereby diminishing OTULIN recruitment to LUBAC and receptor-proximal complexes for local chain editing (10, 11). Importantly, Tyr56 phosphorylation exhibits marked context dependence across distinct stress modalities. During necroptosis, OTULIN undergoes pronounced Tyr56 hyperphosphorylation, which alters RIPK1 ubiquitination dynamics and influences cell death outcomes (36). In a distinct setting, researchers demonstrated that ABL1-dependent OTULIN phosphorylation under genotoxic stress in breast cancer activates Wnt/β-catenin signaling, thereby promoting drug tolerance and metastatic potential (33, 37).

Beyond phosphorylation, OTULIN is subject to non-degradative ubiquitination. The E3 ligase TRIM32 catalyzes K63-linked ubiquitination of OTULIN, which attenuates its binding to LUBAC and consequently enhances NF-κB activation (38). A plausible working model is that TRIM32-mediated K63-linked ubiquitination dynamically uncouples OTULIN from LUBAC by altering the local molecular environment surrounding the HOIP-binding region of OTULIN. Following TNF stimulation, TRIM32 primarily conjugates non-proteolytic K63-linked ubiquitin chains to Lys64 and Lys66, two residues located adjacent to the PIM required for binding the HOIP PUB domain. These ubiquitin chains may sterically occlude the PIM or modify its local conformation and accessibility, thereby weakening the OTULIN–HOIP interaction (38). Another noteworthy regulatory mechanism is proteolytic processing. During apoptosis, caspase-3 cleaves OTULIN at Asp31, and the resulting C-terminal fragment paradoxically exerts a restraining effect on caspase activation and cell death (36). This observation underscores that the post-translational regulation of OTULIN extends beyond canonical covalent modifications to include proteolytic processing as a functionally relevant event. By contrast, whether additional covalent modifications such as SUMOylation or acetylation contribute to the regulation of OTULIN stability, conformation, or substrate repertoire remains largely unexplored and warrants systematic investigation.

## 4 OTULIN-mediated signal regulations: NF-κB, cytokine production, and other signaling pathways

### 4.1 NF-κB

**Figure 2:**
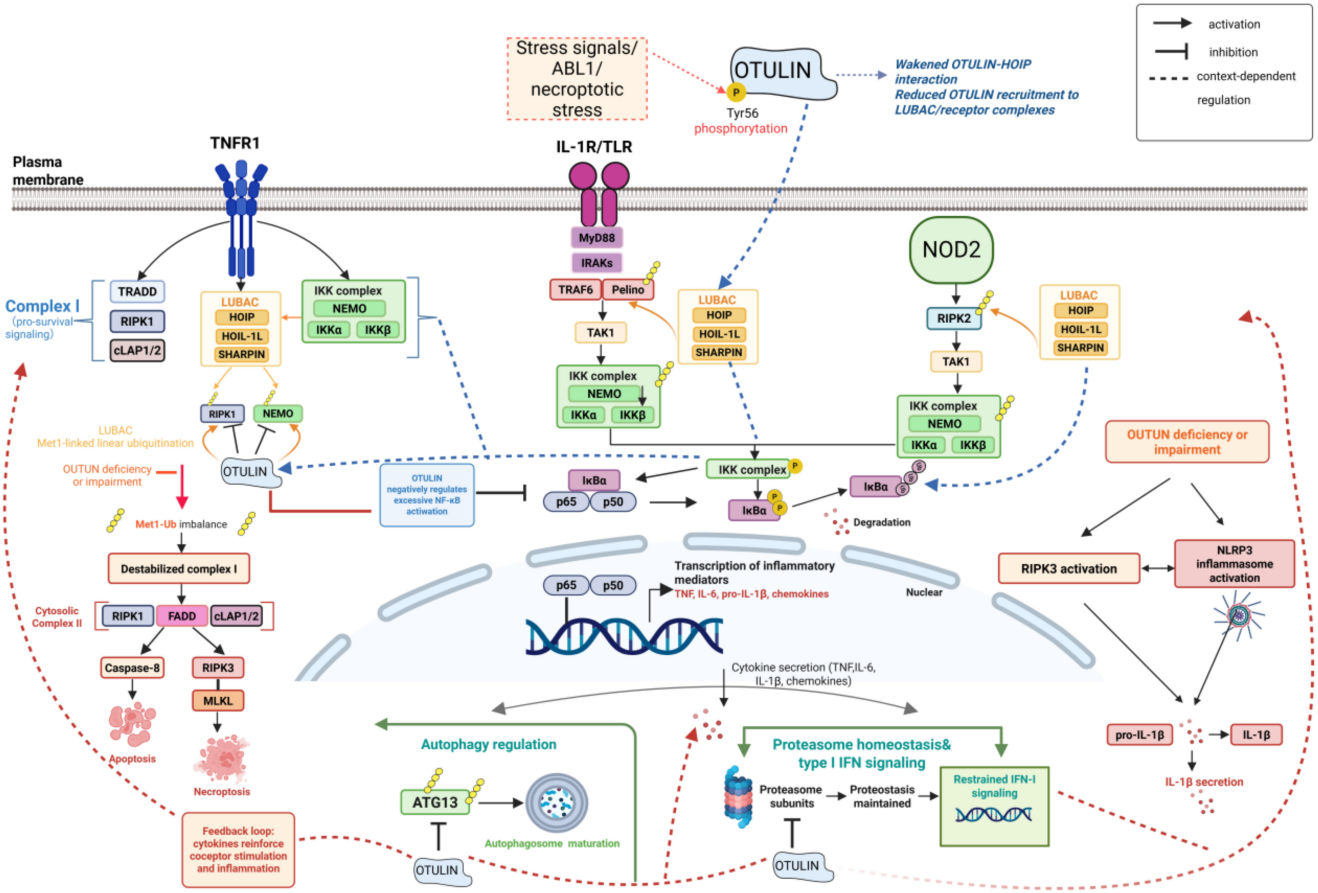
OTULIN-mediated signal regulations. OTULIN maintains Met1-linked ubiquitin homeostasis by counterbalancing LUBAC activity at receptor-proximal signaling complexes. In TNFR1, IL-1R/TLR, and NOD2 pathways, LUBAC-mediated linear ubiquitination promotes IKK recruitment, NF-κB activation, and inflammatory mediator production. OTULIN limits excessive linear ubiquitin accumulation, whereas Tyr56 phosphorylation weakens OTULIN–HOIP binding and reduces OTULIN recruitment to LUBAC-associated complexes. OTULIN deficiency disrupts linear ubiquitin signaling, promotes TNFR1 complex II formation, and enhances apoptosis, necroptosis, inflammasome activation, autophagy dysregulation, proteasomal imbalance, and type I IFN responses, thereby shaping the magnitude and tissue specificity of inflammation.

The core biological function of OTULIN is not simply to shut down inflammatory signaling. Rather, OTULIN operates as a signal editor that fine tunes the abundance, length, spatial distribution, and persistence of linear ubiquitin chains at receptor-proximal sites, thereby governing the stability, lifespan, and ultimate output profile of signaling platforms (**Figure 2**) (13, 39, 40).

Upon stimulation by TNF, IL-1, TLR ligands, or NOD-like receptor agonists, cells rapidly assemble transient signaling platforms at receptor-proximal sites, composed of E3 ubiquitin ligases, deubiquitinases, kinases, and scaffold proteins (13). LUBAC conjugates Met1-linked chains onto NEMO, RIPK1, RIPK2, and other components to facilitate IKK complex recruitment and NF-κB activation, while OTULIN counterbalances this process by preventing aberrant accumulation and undue persistence of linear chains, thus safeguarding against platform over stabilization and inflammatory signal derailment (25, 41, 42). Consequently, the fundamental defect arising from OTULIN deficiency is not merely a failure to terminate inflammatory signaling. Rather, it manifests as a systemic imbalance in receptor-proximal signaling thresholds, signal duration, and downstream inflammatory output.

### 4.2 TNFR1: cell death fate determination

The regulatory role of OTULIN is particularly critical in the TNFR1 pathway (14). Following TNF stimulation, a receptor-proximal complex I assembles around RIPK1, TRADD, cIAP1/2, LUBAC, and the IKK complex (43). Appropriate levels of linear ubiquitination stabilize this platform and promote IKK–NF-κB-mediated pro-survival signaling. When OTULIN is absent, however, aberrant linear ubiquitination of LUBAC components and disrupted Met1-chain homeostasis at the receptor-proximal level destabilize complex I, favoring the formation of cytosolic complex II and the subsequent activation of caspase-8 and RIPK1-dependent cell death (15, 22). Consistent with this model, OTULIN deficiency drives systemic TNF-associated inflammation and promotes FADD/RIPK1-dependent apoptosis in hepatocytes and RIPK1 kinase activity-dependent necroptosis in keratinocytes (21, 44), establishing OTULIN as a critical determinant of the inflammation cell death fate switch (15). An important corollary of this dual role is that catalytically inactive or functionally impaired OTULIN does not invariably result in elevated terminal NF-κB reporter activity. In certain systems, OTULIN dysfunction instead promotes cell death and the consequent release of secondary danger signals, generating more complex, and often more tissue-destructive, inflammatory phenotypes than would be predicted from NF-κB activation alone (45).

### 4.3 Innate immune receptor pathways

OTULIN was reported to play regulatory roles in multiple innate immune receptor pathways (30, 41). In IL-1R/TLR signaling, linear ubiquitin chains similarly participate in the ordered assembly of TRAF6–Pellino–TAK1–IKK signaling nodes (46–49). In the NOD2 pathway, OTULIN has been shown to limit Met1-linked chain accumulation on RIPK2 and associated receptor complex components, thereby restraining excessive downstream signal amplification (13). As a consequence, when OTULIN function is compromised, the host response to PAMPs or DAMPs does not merely become slightly stronger. Instead, it undergoes a compound shift characterized by lowered activation thresholds, prolonged signal duration, and amplified cytokine release, manifested as marked upregulation of TNF, IL-1β, IL-6, and multiple chemokines (14, 35, 45). Clinically, this framework helps explain why patients with OTULIN-associated diseases typically experience fulminant inflammatory flares triggered by infection, tissue injury, or barrier disruption, rather than maintaining a fixed level of chronic hyperinflammation (16).

### 4.4 Cell death and inflammasome

Importantly, OTULIN’s regulation of inflammatory output is not confined to the canonical NF-κB transcriptional axis but is deeply embedded within the coupled network linking cell death, inflammasome activation, and cytokine maturation and release. Recent studies have demonstrated that in myeloid cells, OTULIN deficiency functionally couples RIPK3-dependent cell death with NLRP3 inflammasome activation, markedly enhancing IL-1β maturation and secretion. This augmented IL-1β production does not fully depend on canonical pyroptosis. Instead, RIPK3-driven cell death appears to induce K^+^ efflux, thereby providing the activating conditions for NLRP3 engagement (50). These findings reveal that inflammation in the setting of OTULIN dysfunction is not simply driven by NF-κB-induced transcriptional upregulation of cytokines but is co-shaped by three converging forces, including receptor-proximal ubiquitin platform imbalance, heightened cell death susceptibility, and enhanced inflammasome activation (24).

### 4.5 Non-canonical stress-adaptive pathways

OTULIN also participates in several non-canonical stress-adaptive pathways, further broadening its role as a guardian of inflammatory homeostasis. In the context of autophagy, LUBAC and OTULIN have been shown to co-regulate autophagy initiation and autophagosome maturation through the modulation of ATG13 linear ubiquitination and stability. OTULIN-mediated removal of Met1-linked ubiquitin chains from ATG13 is required to maintain dynamic linear ubiquitin turnover during autophagy. Persistent linear ubiquitination of ATG13 in OTULIN-deficient cells promotes autophagy initiation but impairs autophagosome maturation, indicating that linear ubiquitin homeostasis is essential for efficient autophagic flux. These findings further suggest that linear ubiquitin editing influences both inflammatory receptor platforms and organelle quality control (51). If ATG13-associated Met1-linked ubiquitin chains are required for the initial nucleation event, their prolonged persistence may restrict the dynamic remodeling or stage-specific transition of the ULK1 initiation complex, thereby delaying the progression of the isolation membrane into a mature autophagosome. This model may help explain the dual effect of OTULIN deficiency on autophagy initiation and maturation. With respect to proteostasis and interferon responses, OTULIN removes Met1-linked ubiquitin chains from proteasome subunits to maintain proteasomal function; its deficiency leads to proteasomal imbalance and secondary activation of type I interferon (IFN-I) signaling. This observation demonstrates that OTULIN’s function is beyond the regulation of NF-κB activation intensity to simultaneously connect inflammatory transcription, autophagic flux, protein degradation homeostasis, and IFN responses (16, 19).

In summary, OTULIN functions as the central editor of the linear ubiquitin-dependent inflammatory network. It maintains dynamic equilibrium across TNFR1, IL-1R, TLR, and NOD2 signaling platforms by restraining aberrant Met1-linked chain accumulation at receptor-proximal sites, while simultaneously modulating cell survival and death fate decisions, inflammasome activation, autophagy, and proteasomal homeostasis to collectively determine the intensity, duration, and tissue specificity of inflammatory output.

## 5 Specific roles of OTULIN in regulating the interactions between cells and their microenvironment

**Figure 3:**
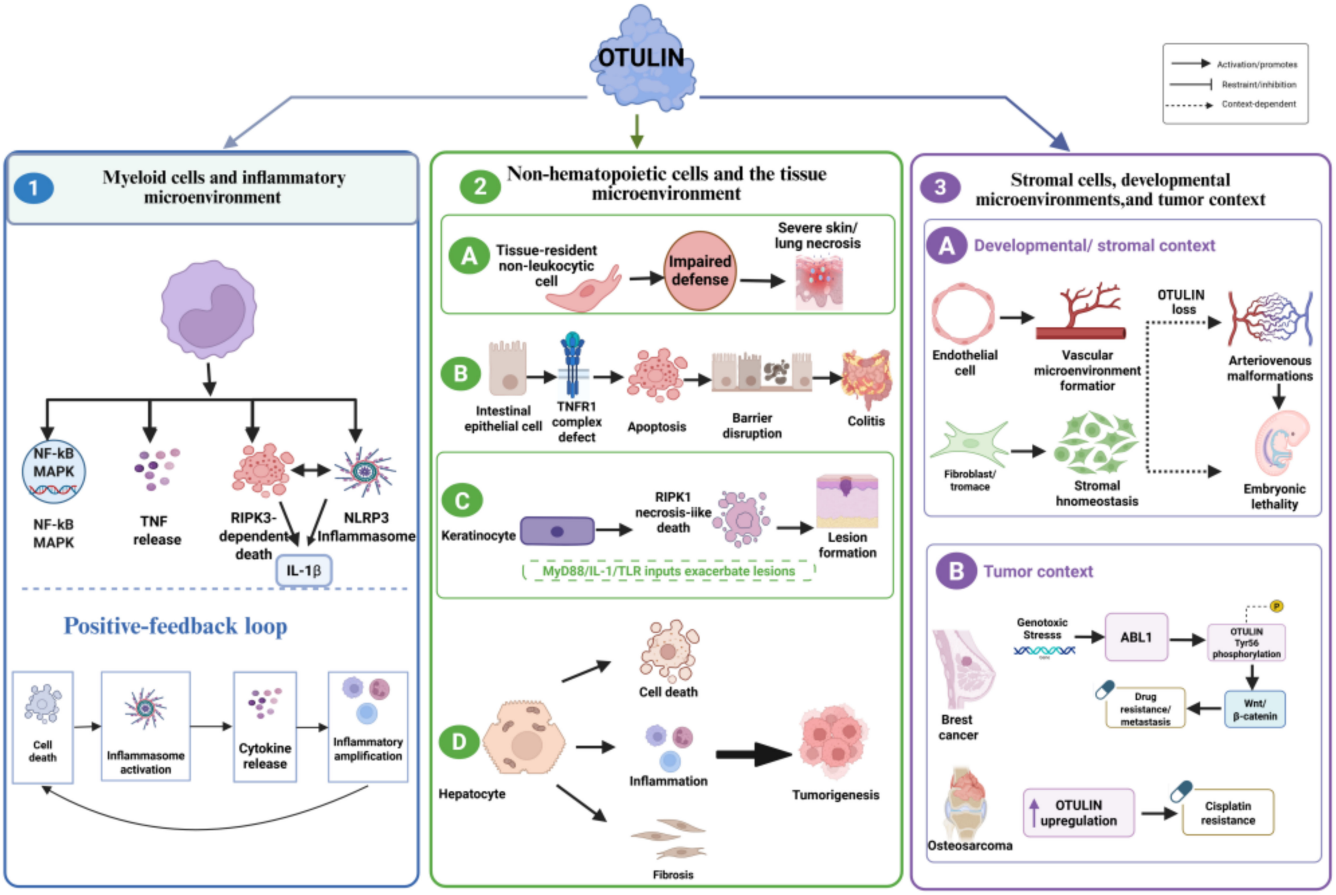
Context-dependent functions of OTULIN in cell-microenvironment homeostasis. OTULIN maintains cell-microenvironment homeostasis across myeloid, non-hematopoietic, stromal, developmental, and tumor settings. In myeloid cells, OTULIN limits NF-κB/MAPK signaling, RIPK3-dependent cell death, NLRP3 inflammasome activation, and cytokine amplification. In tissue-resident non-hematopoietic cells, it preserves epithelial, keratinocyte, and hepatocyte integrity by restraining TNF-driven cell death, barrier disruption, fibrosis, and tumorigenesis. In stromal and developmental contexts, OTULIN supports vascular and tissue microenvironmental integrity, whereas its loss causes vascular malformations and developmental defects. In tumors, OTULIN dysregulation may promote therapy resistance and metastatic adaptation. Collectively, OTULIN dysfunction links tissue fragility to inflammatory amplification, driving context-specific pathology.

In recent years, the development of new biological techniques such as the genetic editing, animal model, muti-omics analysis has fundamentally advanced our understanding of OTULIN biological functions well beyond its traditional molecular definition as a linear deubiquitinase (**Figure 3**) (29). Accumulating evidence indicates that the critical function of OTULIN lies not merely in the removal of Met1-linked ubiquitin chains per se, but in reshaping the modes of interaction between different cell types and their local microenvironments. This OTULIN-dependent crosstalk operates broadly across myeloid cells and the inflammatory milieu, barrier and parenchymal cells and the tissue microenvironment, and endothelial or tumor associated cells and their adaptive niches (20). Accordingly, the pathological consequences of OTULIN deficiency should not be reduced to enhanced inflammatory responses or increased cell death susceptibility within a single cell type. Rather, they represent a broader disruption of cell microenvironment homeostasis that alters both the threshold at which cells sense extrinsic stimuli, including infection, toxins, cytokines, and tissue damage, and the manner in which they respond to local cytokine networks, death signals, and tissue homeostatic feedback (22, 23).

### 5.1 Myeloid cells and inflammatory microenvironment

Within the myeloid compartment, OTULIN functions as a critical gatekeeper that sets the threshold for inflammatory amplification. Early studies established that OTULIN deficiency drives TNF-associated systemic inflammation, and that myeloid-specific deletion alone recapitulates several hallmark features of ORAS in mice, identifying monocytes and macrophages not as passive responders but as primary drivers of inflammation (14, 52). Subsequent work revealed that OTULIN-deficient macrophages not only exhibit enhanced NF-κB/MAPK signaling and spontaneous TNF release but also undergo functional coupling between RIPK3-dependent cell death and NLRP3 inflammasome activation, culminating in amplified IL-1β secretion (45). Mechanistically, OTULIN deficiency licenses RIPK3-mediated cell death to serve as an activating signal for NLRP3, thereby establishing a cell death–inflammasome–cytokine release amplification circuit (50). Consistent with these findings, the hyperinflammatory phenotype observed in LPS-challenged *Otulin*^+/− mice is predominantly driven by CD64^+^ monocytes and macrophages (14). Together, these observations demonstrate that within the myeloid system, OTULIN’s central role is not simply to dampen individual signaling pathways but to prevent positive feedback among necrotic death signals, inflammasome activation, and proinflammatory cytokines at the microenvironmental level, thereby averting the escalation of local inflammation into systemic amplification.

### 5.2 Non-hematopoietic cells and the tissue microenvironment

Non-hematopoietic cells and their tissue microenvironments constitute a critical determinant of clinical phenotype. The most illustrative examples come from human OTULIN haploinsufficiency and a spectrum of conditional knockout models. Studies have shown that the susceptibility of OTULIN-haploinsufficient patients to severe staphylococcal skin and pulmonary necrosis does not primarily arise from leukocyte-intrinsic dysregulation. Instead, it reflects impaired cell-intrinsic defense of non-leukocytic, tissue-resident cells against *Staphylococcus aureus* α-toxin. Complementary mouse models have further revealed a tight coupling between stimulus-triggered local necrosis and systemic inflammation (23). A similar paradigm applies in the intestine, where OTULIN-deficient epithelial cells exhibit heightened TNF sensitivity characterized by impaired TNFR1 complex I assembly, defective LUBAC recruitment, and enhanced cytosolic complex II formation, ultimately leading to increased epithelial apoptosis, barrier disruption, and exacerbated experimental colitis (22). In the skin, keratinocyte-specific *Otulin* deletion induces inflammatory lesions, stem cell identity disruption, and tissue damage marked by RIPK1-dependent necrosis-like cell death. Notably, lesion formation is partially dependent on MyD88 signaling, suggesting that microbe-associated or IL-1/TLR-axis inflammatory inputs further exacerbate the phenotype (20, 44). In the liver, hepatocyte-specific OTULIN loss drives cell death, inflammation, fibrosis, and ultimately tumorigenesis (21, 35, 53). These findings collectively support a framework in which OTULIN-associated disease is best understood as a state of concurrent tissue fragility and immune amplification. When epithelial cells, fibroblasts, or other parenchymal cells become more death-prone due to OTULIN deficiency, the resulting tissue destruction not only compromises structural integrity directly but also releases danger-associated molecular patterns that activate and amplify TNF/IL-1 inflammatory axes, thereby remodeling the local microenvironment. In turn, the inflamed microenvironment further promotes immune cell recruitment, cytokine release, and secondary tissue injury, establishing a self-perpetuating cycle of tissue damage, inflammatory amplification, and progressive destruction.

### 5.3 Stromal cells, developmental microenvironments, and tumor context

From the broader perspective of stromal cells and developmental microenvironments, OTULIN’s functions clearly extend beyond classical inflammatory regulation into fundamental biological processes including angiogenesis and tissue morphogenesis (17). Endothelial cell-specific OTULIN deletion results in arteriovenous malformations and embryonic lethality, demonstrating that linear ubiquitin editing is not only essential for preventing signal derailment under inflammatory conditions but also constitutes a requisite regulatory program for normal vascular microenvironment formation (31). This observation illustrates that OTULIN determines not only how cells respond to extrinsic stimuli within an inflammatory milieu but also how they shape and maintain their microenvironment during development and homeostasis. In the tumor context, current evidence on OTULIN has focused predominantly on tumor cell-intrinsic signal transduction, while its role within the intact tumor microenvironment remains incompletely understood. In breast cancer, ABL1-dependent OTULIN phosphorylation promotes Wnt/β-catenin signaling activation following genotoxic stress, enhancing drug resistance and metastatic potential (33). In osteosarcoma, OTULIN upregulation has been linked to cisplatin resistance (34). These findings suggest that OTULIN may contribute to tumor cell adaptation to therapeutic pressure and the adaptive microenvironment. Notably, OTULIN may promote metastatic progression and therapeutic resistance by remodeling the Met1-linked ubiquitination of Wnt pathway components, thereby sustaining β-catenin signaling. If validated, the ABL1–OTULIN–Wnt/β-catenin axis may extend beyond breast cancer and represent a broader adaptive mechanism through which WNT-active tumors withstand genotoxic therapy. Future studies should identify the specific Wnt-related substrates targeted by OTULIN and determine whether this signaling axis is similarly activated across other tumor types and drug-tolerant cell populations. Whether OTULIN additionally exerts broader microenvironmental effects, through modulation of immune cell infiltration, fibroblast states, or tumor-associated vascular ecology, remains an open question that lacks direct mechanistic evidence (29).

Viewed through the cell–microenvironment interaction, the focus of OTULIN research has shifted from defining its ubiquitin-chain specificity to understanding how it governs the translation of microenvironmental cues into distinct cellular outcomes across different cell types which implies that future intervention strategies must move beyond single-pathway inhibition toward approaches stratified by cell type and tissue context.

## 6 Clinical relevance with dysregulation of OTULIN

The clinical identification of OTULIN-related disease initially stemmed from the analysis of mutation sites in patients with unexplained, severe early-onset autoinflammation. Genetic analyses, including whole-exome sequencing and familial segregation analyses, uncovered biallelic hypomorphic or loss-of-function OTULIN variants in unrelated kindreds, implicating defective linear ubiquitin editing as the molecular basis of a previously unrecognized monogenic autoinflammatory disorder (14, 54). Functional studies in patient-derived cells showed that these variants compromised OTULIN abundance or catalytic activity, impaired the removal of Met1-linked ubiquitin chains, and disturbed the homeostatic control of LUBAC-mediated linear ubiquitination (45, 54). Subsequently, by integrating human genetic studies, cellular analyses, and complementary mouse models, researchers established OTULIN as an essential safeguard against systemic inflammation and autoimmunity (14).

Subsequently, the disorder was subsequently termed OTULIN-related autoinflammation syndrome (ORAS), marked by early onset, severe systemic inflammation. Affected individuals typically present in infancy or early childhood with recurrent fevers, panniculitis, pustular or necrotizing skin lesions, diarrhea or intestinal involvement, articular symptoms, failure to thrive, and markedly elevated acute-phase reactants (54, 55). Mechanistically, patient-derived cells exhibit aberrant inflammatory signaling, such as monocytes display augmented TNF signaling and NF-κB activation, whereas in other cell lineages, OTULIN deficiency is accompanied by diminished LUBAC levels, dysregulated TNF responses, and heightened susceptibility to TNF-induced cell death (14). Therefore, OTULIN deficiency in humans is sufficient to breach the threshold of inflammatory tolerance, converting linear ubiquitin imbalance into clinically manifest systemic inflammation.

Importantly, studies have revealed that individuals carrying only a single aberrant OTULIN allele can develop life-threatening skin and pulmonary necrosis upon infectious challenge, particularly following exposure to Staphylococcus aureus. The pathogenic basis in these cases does not primarily involve leukocyte dysregulation. Rather, it reflects a cell-intrinsic impairment in the capacity of non-leukocyte, tissue-resident cells to mount an effective defense against α-toxin (23). This observation indicates that OTULIN-related pathology does not necessarily require classical biallelic loss of function but can instead manifest under specific environmental second-hit conditions (24, 56). Notably, the OTULIN variant p.Cys129Ser retains the ability to bind LUBAC and linear ubiquitin chains yet lacks deubiquitinase activity, thereby promoting M1-Ub chain accumulation, enhanced LUBAC auto-ubiquitination, impaired LUBAC recruitment to the TNFR1 signaling complex, and increased TNF-induced cell death even in heterozygous carriers (24, 57). Accordingly, OTULIN-related disease can no longer be simplistically classified as autosomal recessive OTULIN deficiency but should instead be understood as a continuous genetic spectrum encompassing biallelic hypomorphism, haploinsufficiency, and dominant-negative effects.

Moreover, beyond the panniculitis and inflammatory skin lesions that typify classical ORAS, patients may present with necrotizing cutaneous or pulmonary involvement, gastrointestinal disease, hepatosplenic abnormalities, infection-triggered acute exacerbations, and varying degrees of immune dysregulation (23, 54, 58). Notably, biallelic defects generally confer earlier disease onset and a higher baseline inflammatory burden, whereas haploinsufficiency and dominant-negative cases tend to exhibit more pronounced trigger dependence, manifesting as rapidly progressive necro-inflammatory phenotypes following infection, toxin exposure, trauma, or other environmental insults (23, 24). Collectively, the clinical heterogeneity of OTULIN-related disease might be shaped by the convergence of multiple determinants, including mutation type, protein stability, residual capacity for LUBAC and linear chain binding, cell-type-specific sensitivity to TNF and cell death signaling, and modifying effects of environmental exposures and genetic background.

Immunologically, OTULIN-related disease has increasingly been recognized as a critical node within the crossover spectrum of autoinflammation and primary immune dysregulation. On one hand, its core manifestations remain driven by innate immune hyperactivation and amplification of the TNF/IL-1 axis (14, 45, 50). On the other hand, OTULIN deficiency extends beyond excessive inflammation to encompass compromised cell-intrinsic antimicrobial defense, an enhanced propensity for necrotizing tissue injury, and features resembling immunodeficiency (23). In clinical practice, heightened diagnostic awareness of OTULIN-related disease is therefore warranted in infants presenting with early-onset severe panniculitis, necrotizing skin lesions, persistent high-grade inflammation, and prominent TNF-driven features. Equally, OTULIN should not be prematurely excluded in adults who develop infection or trauma-triggered necrotizing cutaneous or pulmonary disease, even when only a heterozygous variant is identified.

In summary, the human genetic landscape of OTULIN-related disease has evolved from an initially circumscribed entity, a rare, recessively inherited, early-onset autoinflammatory condition, into a substantially more complex spectrum. This spectrum encompasses classical biallelic ORAS, environmentally triggered haploinsufficiency, and dominant-negative variants characterized by catalytic inactivation with preserved binding capacity. Correspondingly, the clinical phenotype has expanded beyond isolated panniculitis and cutaneous inflammation to include necrotizing tissue injury, infection-associated deterioration, and immune dysregulation.

## 7 Therapeutic strategies for OTULIN-associated diseases

The therapeutic landscape of OTULIN-associated diseases is evolving from empirical anti-inflammatory management toward mechanism-guided, stratified intervention. Current strategies can be broadly organized along three complementary axes (**Table 1**). The first centers on pharmacological modulation of TNF and related inflammatory cascades to restrain aberrantly amplified inflammatory output (14). The second pursues allele-specific correction and functional restoration through gene editing, representing a more causally oriented, long-term direction (56). The third involves immune reconstitution, principally allogeneic hematopoietic stem-cell transplantation (HSCT), for severe, refractory cases driven predominantly by hematopoietic cell-intrinsic defects (16). Because individual patients differ in their genetic backgrounds affected cell populations, and dominant pathological axes, the future of OTULIN-targeted therapy will depend not only on new intervention modalities but, more critically, on establishing precision-stratification frameworks grounded in molecular mechanism and tissue context.

**Table 1:** Therapeutic strategies for OTULIN-associated diseases.

Therapeutic strategies	Mechanism/ Intervention	Targets	Potential application scenarios	Stages of development	Key supporting evidence and limitations
Pharmacological modulators	Targeting the cytokine and receptor axis	TNF, IL-1, IL-6, and JAK/STAT signaling	Classic ORAS	Clinical use (anti-TNF), others exploratory	Anti-TNF therapy alleviates ORAS and haploinsufficiency-associated inflammation, no randomized trials
	Targeting the cell death and inflammasome pathways	RIPK1, RIPK3, MLKL, and NLRP3	OTULIN-deficient patients with prominent necrotic tissue injury and heightened sensitivity to TNF-induced cell death	Preclinical	Mouse models implicate RIPK1/RIPK3/MLKL and the RIPK3–NLRP3 axis in inflammation and cell death, no patient trials
	Targeting the linear ubiquitin pathway	HOIP	OTULIN-deficient patients	Early preclinical	HOIPIN-8 inhibits LUBAC/NF-κB signaling. Systemic LUBAC inhibition may exacerbate cell death
Gene editing and precision repair	Replacement with the wild-type allele	Disease-causing OTULIN alleles	Candidate alleles	Proof of concept	Wild-type allele replacement in patient-derived cells supports variant validation, but not therapeutic efficacy
	Allele-specific silencing of the mutant allele	Mutant OTULIN allele or transcript	Dominant-negative mutations	Conceptual stage	Dominant-negative variants cause M1-Ub accumulation and TNF hypersensitivity, supporting allele-specific silencing
	Gene supplementation or precision correction	OTULIN gene deficiency or dysfunctional OTULIN alleles	Haploinsufficiency or biallelic hypomorphic states	Conceptual stage	Genetic studies support gene supplementation or precision correction, no in vivo efficacy or clinical data
Immune reconstitution	Allogeneic HSCT	Disease-driving hematopoietic compartment	ORAS patients with phenotypes predominantly driven by the hematopoietic system	Single clinical case	One patient achieved remission after HSCT but relapsed as donor chimerism declined, benefit may be limited for non-hematopoietic phenotypes

### 7.1 Pharmacological modulators

Pharmacological intervention can be grouped into three tiers. The first tier comprises cytokine and receptor axis inhibitors directed against TNF, IL-1, IL-6, and JAK/STAT signaling (54, 59–61). Among these, TNF blockade represents the most clinically validated strategy for OTULIN-associated diseases. Patients with classic ORAS frequently respond well to infliximab, etanercept, or adalimumab, with documented improvements in fever, panniculitis, cutaneous inflammation, and systemic inflammatory markers (54, 60). Recent case-series syntheses consistently designate TNF blockade as the first-line approach with the strongest evidence base in biallelic OTULIN deficiency (29). The strengths of this strategy lie in its clinical accessibility and the availability of established drug platforms. Its limitation, however, is that it targets downstream effectors rather than correcting the proximal defect in linear ubiquitination.

The second tier targets cell death and inflammasome pathways, including inhibitors of RIPK1, RIPK3, MLKL, and NLRP3 (50). This approach is particularly relevant in settings characterized by prominent necrotic tissue injury and heightened sensitivity to TNF-induced cell death (62). Multiple studies in OTULIN-deficient models have demonstrated that RIPK1/RIPK3-dependent cell death and NLRP3 inflammasome activation synergistically amplify inflammation and drive tissue damage (44, 50), and drug development targeting these pathways in inflammatory diseases has shown promising progress (63, 64).

The third tier, and the one most proximal to the primary defect, involves direct modulation of the linear ubiquitin pathway. It should be emphasized that no direct OTULIN agonist or functional repair agent has yet entered clinical development. HOIPIN-8, a representative HOIP inhibitor, potently suppresses LUBAC-mediated linear ubiquitination and inhibits TNF-α-induced NF-κB activation in cellular models, establishing pharmacological tractability of the linear ubiquitin axis (65). For OTULIN-deficient patients, however, global LUBAC inhibition does not equate to therapy and may, in certain cell types, exacerbate TNFR1 complex destabilization and cell death. Future development along this axis therefore demands cell-type-selective and context-dependent design rather than systemic suppression (66).

### 7.2 Gene editing and precision repair

Gene editing to achieve precision repair of genomic errors offer the closest approximation to true causal correction, yet they remain largely at the proof-of-concept and functional dissection stage. In patient-derived mesenchymal stem cell models, candidate alleles have been replaced with wild-type counterparts to identify the true disease-driving variant (56), which provides critical methodological foundations for future allele-specific correction, base editing, or prime editing. For dominant-negative mutations, a conceptual strategy exists whereby the mutant allele is selectively silenced while wild-type OTULIN is preserved or supplemented. In contrast, haploinsufficiency or biallelic hypomorphic states will more likely require gene supplementation or precision correction (24). Importantly, OTULIN-associated pathology is not confined to the hematopoietic compartment. Non-hematopoietic cells and tissue-resident populations are central to certain phenotypes. A major translational barrier to OTULIN-directed gene therapy is the need to restore an appropriate level of OTULIN activity specifically within the disease-driving cellular compartment rather than achieving nonspecific systemic expression. In OTULIN-associated cutaneous disease, the epidermal barrier restricts vector penetration, and durable correction would require efficient *in vivo* targeting of basal keratinocytes or long-lived epidermal stem cells, whereas local treatment would not address concomitant abnormalities in other organs (20, 23, 67). In inflammatory liver disease, hepatocytes are comparatively accessible to systemic vectors. However, inflammation, fibrosis, and uptake by non-parenchymal cells may alter vector distribution and compromise hepatocyte-selective delivery (21, 68, 69). In hematopoietic-predominant disease, sustained correction would require targeting long-term repopulating hematopoietic stem cells rather than short-lived circulating myeloid cells, although this strategy would not correct defects intrinsic to tissue-resident non-hematopoietic cells (45, 70). Accordingly, tissue- and cell-selective AAV capsids or lipid nanoparticles are likely to be required to improve delivery to disease-driving cells while limiting off-target perturbation of linear ubiquitin signaling. AAV may support durable expression but is constrained by anti-capsid immunity and difficulty in redosing (71, 72), whereas lipid nanoparticles enable modular and transient delivery but may require repeated administration, and efficient systemic delivery to basal epidermal stem cells and long-term repopulating hematopoietic stem cells remains challenging (73, 74). Even if gene editing becomes technically feasible, achieving sufficiently broad and safe tissue delivery might still remain a decisive bottleneck for clinical translation (23).

### 7.3 Immune reconstitution

The most definitive immune-reconstitution strategy remains allogeneic HSCT. It was reported that a ORAS patient achieved complete resolution of inflammatory symptoms following HSCT after failure of multiple immunomodulatory regimens, suggesting potential curative value for severe inflammatory phenotypes driven primarily by the hematopoietic compartment (45). However, the evidence base remains extremely limited and insufficient to extrapolate HSCT as a universal standard-of-care. Moreover, the haploinsufficiency and non-hematopoietic cell-intrinsic vulnerability imply that OTULIN-associated pathology in some patients extends beyond the hematopoietic lineage. HSCT is therefore more likely to be appropriate for phenotypes predominantly driven by the hematopoietic system and may not fully correct disease manifestations governed by the intrinsic fragility of tissue-resident cells in the skin, lung, and intestine (23). Therefore, immune reconstitution in OTULIN-associated diseases will more likely serve as a stratified option for selected patients rather than a universal therapeutic endpoint.

Encouraging but still limited progress has been made in the treatment of ORAS and other OTULIN-associated inflammatory disorders (54, 55), whereas OTULIN-directed therapeutic strategies in cancer remain at an early exploratory stage (33, 34). Because the biological functions of OTULIN appear to be highly dependent on tumor type and cellular context, and its precise roles in tumorigenesis, therapeutic resistance, and microenvironmental adaptation remain incompletely understood, a broadly applicable and clinically safe therapeutic strategy for therapeutic intervention has yet to be established (18, 35). Future studies should therefore prioritize the development of tumor-selective, mechanism-based, and biomarker-guided precision strategies. OTULIN expression, Tyr56 phosphorylation status, GPX4 dependency, and the activity of the Wnt/β-catenin and the linear ubiquitin pathway should be systematically evaluated as candidate biomarkers to identify tumor subgroups most likely to benefit from therapeutic strategies targeting OTULIN-associated vulnerabilities. In summary, therapeutic strategies for OTULIN-associated diseases are transitioning from controlling terminal inflammatory output to pathological-axis-guided stratified intervention. In the near term, TNF blockade will remain the cornerstone of treatment, with pharmacological exploration of RIPK1/NLRP3 and linear ubiquitin pathway modulators offering the most promising prospects. In near future, allele-specific repair, precision editing in patient-derived cells, and stratified immune reconstitution hold the potential to advance OTULIN-associated diseases from controllable to correctable.

## 8 Discussion

The fundamental biological significance of OTULIN beyonds its catalytic activity of cleaving Met1-linked ubiquitin chains. OTULIN functions as a homeostatic editor of linear ubiquitin signaling, constraining aberrant amplification by LUBAC and thereby governing the intensity, duration, and outcome of inflammatory signaling platforms (10). Two foundational studies (12, 13) established that OTULIN exhibits exquisite specificity for Met1 chains and directly antagonizes LUBAC-dependent inflammatory signaling. Subsequent structural analyses revealed that OTULIN engages the PUB domain of HOIP through its N-terminal PIM with high affinity, and that complex formation is further regulated by Tyr56 phosphorylation, demonstrating that OTULIN is not a passive chain scavenger but an actively recruited brake precisely positioned at the site of chain synthesis (10, 11).

Notably, OTULIN-associated pathology has advanced beyond excessive NF-κB activation to encompass a higher-order paradigm of linear ubiquitin network disequilibrium. OTULIN deficiency not only potentiates TNF/NF-κB signaling but also dysregulates cell death, autophagy, and inflammasome activation (45, 50). Different cell types respond to OTULIN loss in distinct ways, indicating that the underlying defect represents a cell-type- and tissue-context-dependent homeostatic imbalance rather than a mono-pathway aberration. Converging evidence from patient and animal studies demonstrates that OTULIN regulates cellular responses to cytokines and tissue injury, enabling the same extracellular stimulus to drive inflammatory amplification in myeloid cells, induce cell death and tissue fragility in non-hematopoietic cells, or promote adaptive remodeling within stromal and tumor-associated cellular compartments (20, 31, 35, 45, 50). Recent investigations have further expanded the disease spectrum to include dominant-negative variants, revealing that OTULIN pathogenicity is not confined to classical biallelic loss-of-function but may encompass more complex modes in which the protein is present yet functionally inactivated (24).

The OTULIN research might be not limited to catalogue additional phenotypes but to construct an integrative framework that bridges molecular mechanism and clinical stratification. The central questions are how OTULIN selectively shapes Met1-linked ubiquitin signaling output across different cellular and tissue contexts, and how linear ubiquitin disequilibrium couples with cell death, autophagic impairment, and inflammasome activation to ultimately determine tissue-specific pathology. Correspondingly, the key to clinical translation is not merely whether therapeutic agents exist, but how to identify the dominant pathological axis in each patient so as to predict who will benefit most from TNF blockade, RIPK1/RIPK3-targeted intervention, HSCT, or future gene correction. Although TNF inhibition remains the most clinically successful strategy, it principally attenuates downstream inflammatory amplification without correcting the upstream chain-editing defect. A more promising direction might lie in integrating single-cell and spatial multi-omics, linkage-specific ubiquitin proteomics, microenvironmental cues and patient-derived model systems to establish a stratification framework organized around variant type—affected cell—microenvironments—dominant pathway—actionable node. By understanding OTULIN within a cross-scale network can the field advance it from a mechanistic molecule to a bona fide precision-medicine target.

## References

[1] Swatek KN Komander D . Ubiquitin modifications. Cell Res. (2016) 26:399–422. doi: 10.1038/cr.2016.39 27012465 PMC4822133

[2] Lee JM Hammarén HM Savitski MM Baek SH . Control of protein stability by post-translational modifications. Nat Commun. (2023) 14:201. doi: 10.1038/s41467-023-35795-8 36639369 PMC9839724

[3] Sheng X Xia Z Yang H Hu R . The ubiquitin codes in cellular stress responses. Protein Cell. (2024) 15:157–90. doi: 10.1093/procel/pwad045 37470788 PMC10903993

[4] Tokunaga F Iwai K . LUBAC, a novel ubiquitin ligase for linear ubiquitination, is crucial for inflammation and immune responses. Microbes Infect. (2012) 14:563–72. doi: 10.1016/j.micinf.2012.01.011 22309894

[5] Tokunaga F Sakata S Saeki Y Satomi Y Kirisako T Kamei K . Involvement of linear polyubiquitylation of NEMO in NF-kappaB activation. Nat Cell Biol. (2009) 11:123–32. doi: 10.1038/ncb1821 19136968

[6] Fiil BK Gyrd-Hansen M . The Met1-linked ubiquitin machinery in inflammation and infection. Cell Death Differ. (2021) 28:557–69. doi: 10.1038/s41418-020-00702-x 33473179 PMC7816137

[7] Gerlach B Cordier SM Schmukle AC Emmerich CH Rieser E Haas TL . Linear ubiquitination prevents inflammation and regulates immune signalling. Nature. (2011) 471:591–6. doi: 10.1038/nature09816 21455173

[8] Wang H Zhang Z Li Y Li S Guan J Liu Z . Mitotic spindle-organizing protein MZT2A promotes cisplatin resistance through NEMO ubiquitination and NF-κB activation. J Biol Chem. (2026) 302:111330. doi: 10.1016/j.jbc.2026.111330 41763311 PMC13049933

[9] Zein L Dietrich M Balta D Bader V Scheuer C Zellner S . Linear ubiquitination at damaged lysosomes induces local NFKB activation and controls cell survival. Autophagy. (2025) 21:1075–95. doi: 10.1080/15548627.2024.2443945 39744815 PMC12013452

[10] Elliott PR Nielsen SV Marco-Casanova P Fiil BK Keusekotten K Mailand N . Molecular basis and regulation of OTULIN-LUBAC interaction. Mol Cell. (2014) 54:335–48. doi: 10.1016/j.molcel.2014.03.018 24726323 PMC4017264

[11] Schaeffer V Akutsu M Olma MH Gomes LC Kawasaki M Dikic I . Binding of OTULIN to the PUB domain of HOIP controls NF-κB signaling. Mol Cell. (2014) 54:349–61. doi: 10.1016/j.molcel.2014.03.016 24726327

[12] Keusekotten K Elliott PR Glockner L Fiil BK Damgaard RB Kulathu Y . OTULIN antagonizes LUBAC signaling by specifically hydrolyzing Met1-linked polyubiquitin. Cell. (2013) 153:1312–26. doi: 10.1016/j.cell.2013.05.014 23746843 PMC3690481

[13] Fiil BK Damgaard RB Wagner SA Keusekotten K Fritsch M Bekker-Jensen S . OTULIN restricts Met1-linked ubiquitination to control innate immune signaling. Mol Cell. (2013) 50:818–30. doi: 10.1016/j.molcel.2013.06.004 23806334 PMC4194427

[14] Damgaard RB Walker JA Marco-Casanova P Morgan NV Titheradge HL Elliott PR . The deubiquitinase OTULIN is an essential negative regulator of inflammation and autoimmunity. Cell. (2016) 166:1215–30.e20. doi: 10.1016/j.cell.2016.07.019 27523608 PMC5002269

[15] Heger K Wickliffe KE Ndoja A Zhang J Murthy A Dugger DL . OTULIN limits cell death and inflammation by deubiquitinating LUBAC. Nature. (2018) 559:120–4. doi: 10.1038/s41586-018-0256-2 29950720

[16] Staels F Bücken L De Vuyst L Willemsen M Van Nieuwenhove E Gerbaux M . OTULIN haploinsufficiency predisposes to environmentally directed inflammation. Front Immunol. (2024) 15:983686. doi: 10.3389/fimmu.2024.983686 38827742 PMC11140568

[17] Rivkin E Almeida SM Ceccarelli DF Juang YC MacLean TA Srikumar T . The linear ubiquitin-specific deubiquitinase gumby regulates angiogenesis. Nature. (2013) 498:318–24. doi: 10.1038/nature12296 23708998 PMC4931916

[18] Li M Li L Asemota S Kakhniashvili D Narayanan R Wang X . Reciprocal interplay between OTULIN-LUBAC determines genotoxic and inflammatory NF-κB signal responses. Proc Natl Acad Sci USA. (2022) 119:e2123097119. doi: 10.1073/pnas.2123097119 35939695 PMC9388121

[19] Tao P Wang S Ozen S Lee PY Zhang J Wang J . Deubiquitination of proteasome subunits by OTULIN regulates type I IFN production. Sci Adv. (2021) 7:eabi6794. doi: 10.1126/sciadv.abi6794 34797715 PMC8604410

[20] Hoste E Lecomte K Annusver K Vandamme N Roels J Maschalidi S . OTULIN maintains skin homeostasis by controlling keratinocyte death and stem cell identity. Nat Commun. (2021) 12:5913. doi: 10.26226/morressier.61081ff8bc981037240fe45a 34625556 PMC8501048

[21] Verboom L Martens A Priem D Hoste E Sze M Vikkula H . OTULIN prevents liver inflammation and hepatocellular carcinoma by inhibiting FADD- and RIPK1 kinase-mediated hepatocyte apoptosis. Cell Rep. (2020) 30:2237–47.e6. doi: 10.1016/j.celrep.2020.01.028 32075762

[22] Verboom L Anderson CJ Jans M Petta I Blancke G Martens A . OTULIN protects the intestinal epithelium from apoptosis during inflammation and infection. Cell Death Dis. (2023) 14:534. doi: 10.1038/s41419-023-06058-7 37598207 PMC10439912

[23] Spaan AN Neehus AL Laplantine E Staels F Ogishi M Seeleuthner Y . Human OTULIN haploinsufficiency impairs cell-intrinsic immunity to staphylococcal α-toxin. Science. (2022) 376:eabm6380. doi: 10.1126/science.abm6380 35587511 PMC9233084

[24] Davidson S Shibata Y Collard S Zheng H Kong K Sun JM . Dominant negative OTULIN-related autoinflammatory syndrome. J Exp Med. (2024) 221:e20222171. doi: 10.1084/jem.20222171 38630025 PMC11022884

[25] Mevissen TE Hospenthal MK Geurink PP Elliott PR Akutsu M Arnaudo N . OTU deubiquitinases reveal mechanisms of linkage specificity and enable ubiquitin chain restriction analysis. Cell. (2013) 154:169–84. doi: 10.1016/j.cell.2013.05.046 23827681 PMC3705208

[26] Stangl A Elliott PR Pinto-Fernandez A Bonham S Harrison L Schaub A . Regulation of the endosomal SNX27-retromer by OTULIN. Nat Commun. (2019) 10:4320. doi: 10.1038/s41467-019-12309-z 31541095 PMC6754446

[27] Smit JJ Monteferrario D Noordermeer SM van Dijk WJ van der Reijden BA Sixma TK . The E3 ligase HOIP specifies linear ubiquitin chain assembly through its RING-IBR-RING domain and the unique LDD extension. EMBO J. (2012) 31:3833–44. doi: 10.1038/emboj.2012.217 22863777 PMC3463842

[28] Elliott PR Komander D . Regulation of Met1-linked polyubiquitin signalling by the deubiquitinase OTULIN. FEBS J. (2016) 283:39–53. doi: 10.1111/febs.13547 26503766 PMC4765238

[29] Dou B Jiang G Peng W Liu C . OTULIN deficiency: focus on innate immune system impairment. Front Immunol. (2024) 15:1371564. doi: 10.3389/fimmu.2024.1371564 38774872 PMC11106414

[30] Weinelt N van Wijk SJL . Ubiquitin-dependent and -independent functions of OTULIN in cell fate control and beyond. Cell Death Differ. (2021) 28:493–504. doi: 10.1038/s41418-020-00675-x 33288901 PMC7862380

[31] Fu Y Wang H Dai H Zhu Q Cui CP Sun X . OTULIN allies with LUBAC to govern angiogenesis by editing ALK1 linear polyubiquitin. Mol Cell. (2021) 81:3187–204.e7. doi: 10.1016/j.molcel.2021.05.031 34157307

[32] Quoc QL Kim Y Park G Cao TBT Choi Y Park YH . Downregulation of otulin induces inflammasome activation in neutrophilic asthma. J Allergy Clin Immunol. (2024) 154:557–70. doi: 10.1016/j.jaci.2024.03.021 38599290

[33] Wang W Li M Ponnusamy S Chi Y Xue J Fahmy B . ABL1-dependent OTULIN phosphorylation promotes genotoxic Wnt/β-catenin activation to enhance drug resistance in breast cancers. Nat Commun. (2020) 11:3965. doi: 10.1038/s41467-020-17770-9 32770022 PMC7414915

[34] Zheng Z Zeng Y Bao X Huang C Guo F Xu F . OTULIN confers cisplatin resistance in osteosarcoma by mediating GPX4 protein homeostasis to evade the mitochondrial apoptotic pathway. J Exp Clin Cancer Res. (2024) 43:330. doi: 10.1186/s13046-024-03249-8 39721999 PMC11670407

[35] Damgaard RB Jolin HE Allison MED Davies SE Titheradge HL McKenzie ANJ . OTULIN protects the liver against cell death, inflammation, fibrosis, and cancer. Cell Death Differ. (2020) 27:1457–74. doi: 10.1038/s41418-020-0532-1 32231246 PMC7206033

[36] Douglas T Saleh M . Post-translational modification of OTULIN regulates ubiquitin dynamics and cell death. Cell Rep. (2019) 29:3652–63.e5. doi: 10.1016/j.celrep.2019.11.014 31825842

[37] Wang W Wu ZH . OTULIN couples WNT signaling to resistance in triple-negative breast cancer. Mol Cell Oncol. (2020) 7:1825904. doi: 10.1080/23723556.2020.1825904 33241111 PMC7671033

[38] Zhao M Song K Hao W Wang L Patil G Li Q . Non-proteolytic ubiquitination of OTULIN regulates NF-κB signaling pathway. J Mol Cell Biol. (2020) 12:163–75. doi: 10.1093/jmcb/mjz081 31504727 PMC7181720

[39] Hrdinka M Gyrd-Hansen M . The met1-linked ubiquitin machinery: emerging themes of (De)regulation. Mol Cell. (2017) 68:265–80. doi: 10.1016/j.molcel.2017.09.001 29053955

[40] Verboom L Hoste E van Loo G . OTULIN in NF-κB signaling, cell death, and disease. Trends Immunol. (2021) 42:590–603. doi: 10.1016/j.it.2021.05.003 34074601

[41] Fiil BK Gyrd-Hansen M . Met1-linked ubiquitination in immune signalling. FEBS J. (2014) 281:4337–50. doi: 10.1111/febs.12944 25060092 PMC4286102

[42] Iwai K . LUBAC-mediated linear ubiquitination: a crucial regulator of immune signaling. Proc Jpn Acad Ser B Phys Biol Sci. (2021) 97:120–33. doi: 10.2183/pjab.97.007 33692228 PMC8019854

[43] Dondelinger Y Darding M Bertrand MJ Walczak H . Poly-ubiquitination in TNFR1-mediated necroptosis. Cell Mol Life Sci. (2016) 73:2165–76. doi: 10.1007/s00018-016-2191-4 27066894 PMC4887548

[44] Schünke H Göbel U Dikic I Pasparakis M . OTULIN inhibits RIPK1-mediated keratinocyte necroptosis to prevent skin inflammation in mice. Nat Commun. (2021) 12:5912. doi: 10.1038/s41467-021-25945-1 34625557 PMC8501112

[45] Damgaard RB Elliott PR Swatek KN Maher ER Stepensky P Elpeleg O . OTULIN deficiency in ORAS causes cell type-specific LUBAC degradation, dysregulated TNF signalling and cell death. EMBO Mol Med. (2019) 11:e9324. doi: 10.15252/emmm.201809324 30804083 PMC6404114

[46] Emmerich CH Ordureau A Strickson S Arthur JS Pedrioli PG Komander D . Activation of the canonical IKK complex by K63/M1-linked hybrid ubiquitin chains. Proc Natl Acad Sci USA. (2013) 110:15247–56. doi: 10.1073/pnas.1314715110 23986494 PMC3780889

[47] Strickson S Emmerich CH Goh ETH Zhang J Kelsall IR Macartney T . Roles of the TRAF6 and Pellino E3 ligases in MyD88 and RANKL signaling. Proc Natl Acad Sci USA. (2017) 114:E3481–e9. doi: 10.1073/pnas.1702367114 28404732 PMC5410814

[48] Cohen P . The TLR and IL-1 signalling network at a glance. J Cell Sci. (2014) 127:2383–90. doi: 10.1242/jcs.149831 24829146 PMC4038938

[49] Tang RC Tang Z Yu SS Zhang A Geng S Yu H . Deubiquitinase OTULIN dampens RIG-I-dependent antiviral signaling by removing linear ubiquitination from TRAF6. Proc Natl Acad Sci USA. (2026) 123:e2517201123. doi: 10.1073/pnas.2517201123 41802043 PMC12994173

[50] Doglio MG Verboom L Ruilova Sosoranga E Frising UC Asaoka T Gansemans Y . Myeloid OTULIN deficiency couples RIPK3-dependent cell death to Nlrp3 inflammasome activation and IL-1β secretion. Sci Immunol. (2023) 8:eadf4404. doi: 10.1126/sciimmunol.adf4404 38000038

[51] Chu Y Kang Y Yan C Yang C Zhang T Huo H . LUBAC and OTULIN regulate autophagy initiation and maturation by mediating the linear ubiquitination and the stabilization of ATG13. Autophagy. (2021) 17:1684–99. doi: 10.1080/15548627.2020.1781393 32543267 PMC8354604

[52] Wang Q Wang L Botchway BOA Zhang Y Huang M Liu X . OTULIN can improve spinal cord injury by the NF-κB and Wnt/β-catenin signaling pathways. Mol Neurobiol. (2024) 61:8820–30. doi: 10.1007/s12035-024-04134-3 38561559

[53] Gao L Chang X Han Y Li J Meng Y Yang X . OTULIN orchestrates NCOA4-FTH1 complex to alleviate APAP-induced hepatocyte ferroptosis. Int Immunopharmacol. (2025) 154:114490. doi: 10.1016/j.intimp.2025.114490 40158433

[54] Zhou Q Yu X Demirkaya E Deuitch N Stone D Tsai WL . Biallelic hypomorphic mutations in a linear deubiquitinase define otulipenia, an early-onset autoinflammatory disease. Proc Natl Acad Sci USA. (2016) 113:10127–32. doi: 10.1073/pnas.1612594113 27559085 PMC5018768

[55] Arts RJW van der Linden TJ van der Made CI Hendriks MMC van der Heijden WA de Mast Q . OTULIN haploinsufficiency-related fasciitis and skin necrosis treated by TNF inhibition. J ClinImmunol. (2023) 44:10. doi: 10.1007/s10875-023-01630-4 38129331

[56] Takeda Y Ueki M Matsuhiro J Walinda E Tanaka T Yamada M . A de novo dominant-negative variant is associated with OTULIN-related autoinflammatory syndrome. J Exp Med. (2024) 221:e20231941. doi: 10.1084/jem.20231941 38652464 PMC11040501

[57] Elbæk CR Gradinaru S Dahlström AM Frueh A Jahan AS Cuenco J . M1-linked ubiquitination by LUBAC regulates AMPK signalling and the response to energetic stress. Cell Death Differ. (2026). doi: 10.1038/s41418-026-01675-z PMC13434424 41688728

[58] Caballero-Oteyza A Crisponi L Peng XP Wang H Mrovecova P Olla S . OTULIN-related conditions: Report of a new case and review of the literature using GenIA. Clin Immunol. (2024) 265:110292. doi: 10.1016/j.clim.2024.110292 38914362

[59] Blackstone SA Kastner DL Broderick L . Autoinflammatory syndromes: Updates in management. J Allergy Clin Immunol. (2024) 153:85–9. doi: 10.1016/j.jaci.2023.11.001 37926121

[60] Du Y Liu M Nigrovic PA Dedeoglu F Lee PY . Biologics and JAK inhibitors for the treatment of monogenic systemic autoinflammatory diseases in children. J Allergy Clin Immunol. (2023) 151:607–18. doi: 10.1016/j.jaci.2022.12.816 36707349 PMC9992337

[61] Koga T . Targeting cytokine pathways: the role of biologics in autoinflammatory disorders. Expert Rev Clin Immunol. (2026) 22:107–21. doi: 10.1080/1744666x.2026.2625277 41620931

[62] Koerner L Li X Silnov E Laurien L Pasparakis M . RIPK1 autophosphorylation at S161 mediates cell death and inflammation. J Exp Med. (2025) 222:e20250279. doi: 10.1084/jem.20250279 40996439 PMC12462663

[63] Sun ALA Gillies JD Shen Y Deng H Xue F Ma Y . A phase I randomized study to evaluate safety, pharmacokinetics, and pharmacodynamics of SIR2446M, a selective RIPK1 inhibitor, in healthy participants. Clin Transl Sci. (2024) 17:e13857. doi: 10.1111/cts.13857 38949195 PMC11215690

[64] Ramalingam V . NLRP3 inhibitors: unleashing their therapeutic potential against inflammatory diseases. Biochem Pharmacol. (2023) 218:115915. doi: 10.1016/j.bcp.2023.115915 37949323

[65] Katsuya K Oikawa D Iio K Obika S Hori Y Urashima T . Small-molecule inhibitors of linear ubiquitin chain assembly complex (LUBAC), HOIPINs, suppress NF-κB signaling. Biochem Biophys Res Commun. (2019) 509:700–6. doi: 10.1016/j.bbrc.2018.12.164 30611571

[66] Fuseya Y Iwai K . Biochemistry, pathophysiology, and regulation of linear ubiquitination: intricate regulation by coordinated functions of the associated ligase and deubiquitinase. Cells. (2021) 10:2706. doi: 10.3390/cells10102706 34685685 PMC8534859

[67] Hirsch T Rothoeft T Teig N Bauer JW Pellegrini G De Rosa L . Regeneration of the entire human epidermis using transgenic stem cells. Nature. (2017) 551:327–32. doi: 10.1038/nature24487 29144448 PMC6283270

[68] Ferriero R Bruno G Padula A Pisano S Boffa I Gargaro M . Impact of liver fibrosis on AAV-mediated gene transfer to mouse hepatocytes. Nat Commun. (2025) 16:2118. doi: 10.1038/s41467-025-57382-9 40064861 PMC11893804

[69] Kim JJ Kurial SNT Choksi PK Nunez M Lunow-Luke T Bartel J . AAV capsid prioritization in normal and steatotic human livers maintained by machine perfusion. Nat Biotechnol. (2025) 43:1966–78. doi: 10.1038/s41587-024-02523-6 39881029 PMC12304247

[70] Newby GA Yen JS Woodard KJ Mayuranathan T Lazzarotto CR Li Y . Base editing of haematopoietic stem cells rescues sickle cell disease in mice. Nature. (2021) 595:295–302. doi: 10.1038/s41586-021-03609-w 34079130 PMC8266759

[71] Reiss UM Davidoff AM Tuddenham EGD Chowdary P McIntosh J Muczynski V . Sustained clinical benefit of AAV gene therapy in severe hemophilia B. N Engl J Med. (2025) 392:2226–34. doi: 10.1056/nejmoa2414783 40499172 PMC7617823

[72] Manno CS Pierce GF Arruda VR Glader B Ragni M Rasko JJ . Successful transduction of liver in hemophilia by AAV-Factor IX and limitations imposed by the host immune response. Nat Med. (2006) 12:342–7. doi: 10.1038/nm1358 16474400

[73] Cheng Q Wei T Farbiak L Johnson LT Dilliard SA Siegwart DJ . Selective organ targeting (SORT) nanoparticles for tissue-specific mRNA delivery and CRISPR-Cas gene editing. Nat Nanotechnol. (2020) 15:313–20. doi: 10.1038/s41565-020-0669-6 32251383 PMC7735425

[74] Pardi N Tuyishime S Muramatsu H Kariko K Mui BL Tam YK . Expression kinetics of nucleoside-modified mRNA delivered in lipid nanoparticles to mice by various routes. J Control Release. (2015) 217:345–51. doi: 10.1016/j.jconrel.2015.08.007 26264835 PMC4624045

